# First Report of Fascioliasis of Ruminants in the Kharaa River Basin and Identification of Snail Hosts in Mongolia

**DOI:** 10.1155/vmi/6226110

**Published:** 2026-02-06

**Authors:** Lkhagvatseren Sukhbaatar, Nora G. Cleary, Davaajargal Tserennyam, Enkhjargal Enkherdene, Chinchuluun Boldbaatar, Gantuya Sambuu, Munkhjargal Tserendorj, Otgonpurev Sukhbaatar, Batsukh Zayat, Michael E. von Fricken

**Affiliations:** ^1^ Institute of Veterinary Medicine, Zaisan, Ulaanbaatar, 17029, Mongolia; ^2^ One Health Center of Excellence, Department of Environmental and Global Health, University of Florida, Gainesville, 32610, Florida, USA, ufl.edu; ^3^ National Cancer Center of Mongolia, School of Medicine, Mongolian National University of Medical Sciences, Ulaanbaatar, 14210, Mongolia, mnums.edu.mn; ^4^ School of Applied Science, Mongolian State University of Life Sciences, Zaisan, Ulaanbaatar, 17029, Mongolia

**Keywords:** fascioliasis, fecal egg, liver fluke, livestock, snail

## Abstract

Fascioliasis is a parasitic liver disease of mammals induced by liver flukes, *Fasciola hepatica* and *Fasciola gigantica. Fasciola* spp. rely on their definitive hosts, ruminants, and intermediate hosts, snails, to survive and can incidentally infect humans as definitive hosts. Ruminant (goat, sheep, and cattle) liver and fecal samples were collected from the Kharaa River Basin (KRB) of Mongolia during 2018–2020. A total of 807 adult liver flukes were found in livers of 18 goats, 21 sheep, and 1 cattle, with morphological identification of *F. hepatica* species. A total of 350 fecal samples selected using a “risk‐based surveillance” method in the KRB were tested for *Fasciola* spp. eggs with 50.3% (*n* = 151/350) positive. By animal, cattle had the highest percentage of *Fasciola* spp. positive samples of 70% (14/20), followed by sheep with 49.4% (87/176) and goats with 32.5% (50/154). Six‐hundred *Lymnaied* spp. snails, an intermediate host of *Fasciola*, were obtained from 18 locations in six provinces and one provincial municipality and morphometrically identified as *Radix bactriana* (94.8%) and *Lymnaea stagnalis* (5.2%). This study represents the first report of fascioliasis in indigenous animals in Mongolia and when paired with the detected prevalence of *Fasciola* spp. eggs in feces, suggests endemic circulation in the KRB region.

## 1. Introduction

Fascioliasis is a parasitic liver disease of mammals induced by liver flukes, mainly *Fasciola hepatica* or *Fasciola gigantica* species [1]. *Fasciola* has an estimated human prevalence of 4.5%–5% worldwide with data from over 81 countries but there is a large data gap in many countries including Mongolia [2, 3]. This zoonotic disease can be detrimental for livestock productivity and lead to fatalities, causing a huge economic burden for pastoralists [4]. Mongolia is sparsely populated but livestock dense with 71.1 million livestock reported in 2022 where over 98% of livestock are managed under traditional pastoral animal husbandry [5, 6]. However, pastoralists face many hardships that are exacerbated by environmental stressors driven by climate change, the high burden of infectious disease in livestock, and limited access to therapeutics and novel technologies that could improve the overall health of herds and herders’ livelihoods. About 26% of the Mongolian population practices animal husbandry in proximity to livestock [7, 8].

These populations are at an increased risk of *Fasciola* infection through the consumption of contaminated food and water, especially in areas where a high percentage of infected ruminants reside, potentially shedding flukes in their stool [4]. In humans, fascioliasis infection caused by *F. gigantica* or *F. hepatica* commonly presents acutely as fever, nausea, abdominal pain, and elevation of liver enzymes, potentially leading to a long‐term infection. *Fasciola* spp. require two hosts to complete their lifecycle: ruminants and snails [9]. As definitive hosts, ruminants provide an environment for adult flukes to reproduce [10], whereas intermediate snail hosts in the Lymnaeidae family provide a suitable habitat for trematodes to multiply [4].

A 2016 report morphologically identified *F. hepatica* in liver flukes (*n* = 5–151) from slaughtered animals (sheep *n* = 2, goats *n* = 5, and cattle *n* = 1) in the Mandal soum of Selenge province in Mongolia. Prior to this, there had been only two registered imported cases of bovine fascioliasis in Mongolia in 1967 and 1981 from Hungary and Russia, respectively [11–13]. The Mandal soum is in the Kharaa River Basin (KRB) in northern Mongolia with a rich river network, originating in the Khentii mountains where the temperature and water availability for trematode eggs and metacercarial development and survival reach its peak from April to October. This results in heightened snail activity during the warmer, drier summer months compared with the cold winters where snail populations are dormant [14].

The environmental conditions, presence of livestock hosts, intermediate snail hosts, and incidental human hosts present a One Health risk for *Fasciola* spp. in this region. Fascioliasis is critical to track, as zoonotic cases can be prevented with proper preventative measures, maintenance of animal health, and prevention of imported infected animals. Speciating *Fasciola* through morphological studies is valuable although less reliable than molecular confirmation, as it can guide prevention and control measures, while contributing to our understanding of disease ecology and clinical presentation in both animals and humans [15, 16]. This study aimed to determine the prevalence of *Fasciola* spp. in ruminants and identify potential snail intermediate hosts in KRB.

## 2. Materials and Methods

### 2.1. Study Area

**FIGURE 1:**
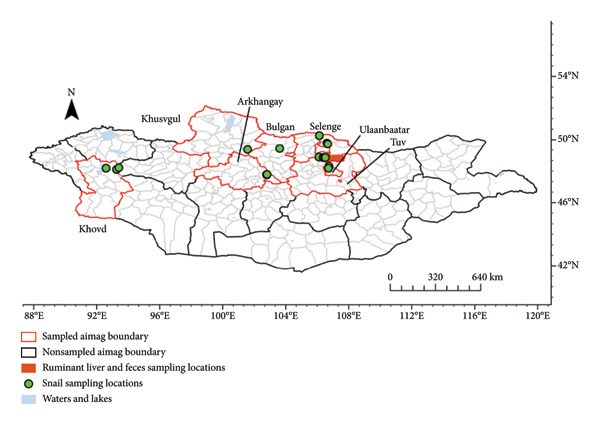
Collection sites of ruminant liver and fecal samples (county level) shown in red in Selenge and coordinate locations for freshwater snails in Mongolia from 2018 to 2020 in six provinces and one provincial municipality.

The study area for ruminant liver and fecal samples was in Mandal soum (county) in Selenge province in the KRB of northern Mongolia. Snail samples were collected from rivers and lakes in six provinces including Khusvgul, Khovd, Arhangay, Bulgan, Tuv, and Selenge and one provincial municipality, Ulaanbaatar (Figure 1).

### 2.2. Study Design and Sampling

Liver samples were collected from 40 slaughtered ruminants (sheep *n* = 21, goat *n* = 18, and cattle *n* = 1) from October to December in 2018, 2019, and 2020 in Mandal, Selenge. The animals which ranged from one to eight years old were grouped into young (one to three years old) and adult ( four to eight years old) [17]. In the same region, fecal samples were collected from 350 herds of at‐risk sheep, goat, and cattle, collected from May to September of each respective year (Table 1). The at‐risk population included any animals showing clinical symptoms for parasitic infections in KRB. The criteria for clinical symptoms was based on eye, hair, motion, eye mucous color, and anemia chart (FAMACHA‐FAffa Malan CHArt) ≥ 3, and body condition score (BCS) ≤ 2 [18]. Six‐hundred lymnaeid snail samples were collected from six locations in the Kharaa river during three visits in 2018–2020 and 12 locations in the Eroo and Selenge rivers and Khar and Ugii lakes during single visits in 2018–2020. The target area was visited six times and control areas were visited once. Sampling sites were selected by local professionals known to have snails present. Snail samples were gathered by hand from the bottom of a sieve and stored in groups of ten by location in sterile vials with water. Following morphological identification, pooled snails were placed in vials with 70% ethanol for fixation and morphometric analysis.

**TABLE 1:** Ruminant sampling scheme for the at‐risk population in the target area for fecal sample collection sorted by collection year and host animal.

Year	Sheep		Goat		Cattle		Total
	Young	Adult	Young	Adult	Young	Adult	
2018	50	30	40	30	—	—	150
2019	30	20	22	20	6	2	100
2020	24	22	24	18	6	6	100
Total	104	72	86	68	12	8	350

All sampling was conducted in accordance with the regulations of the Experimental Animal Ethics Committee of the Mongolian State University of Life Sciences, Mongolia (accession number Vet.SS:19/01/15) and the Fundamental Guidelines under the Jurisdiction of the Mongolian Foundation for Science and Technology, Ministry of Education and Science of Mongolia.

### 2.3. Laboratory Analysis

The FAO protocol was followed for helminth parasite diagnosis of ruminants [19]. Adult flukes were isolated from livers of ruminants using the National Standard for Helminthological Necropsy in Ruminants (MNS6471:2014). Flukes were halved with half stored in 70% ethanol and half washed in rinse water overnight for relaxation, dried completely, stained for morphometric patterns, and measured using a morphometric key [20]. Measurements of adult *Fasciola* spp. were taken using a stereo microscope SMZ645 including body width (BW), body length (BL), ratio of BL to BW (BL/BW), distance between the ventral sucker and the posterior end (VS‐P), and distance between the vitelline glands and the posterior end of the body (Vit‐P).

Upon collection, fecal samples were stored at 4°C and analyzed within one week of collection. *Fasciola* spp. eggs were identified using the classic sedimentation method observing the trematode egg using Shinova BN‐800M microscopy’s bar measurement in ruminant feces and the prevalence of fascioliasis was based on *Fasciola* spp. egg positive samples. Lymnaied snails were morphometrically identified using shell measurements performed by ocular‐micrometer (accuracy ±0.1 mm) using appropriate keys [21]. The stereomicroscope was calibrated using a standard micrometer scale prior to observation of flukes with a measurement error range of ±1.0 mm. Replicate measurements were taken for uncertain samples to ensure accuracy. Maps were created for liver, feces, and snail sampling locations based on GPS coordinates using ArcGIS Pro 3.2.0 [22]. Shapefiles were obtained from https://data.humdata.org/dataset/cod-ab-mng and https://data.humdata.org/dataset/mongolia-water-bodies.

## 3. Results

### 3.1. Liver Flukes

**FIGURE 2:**
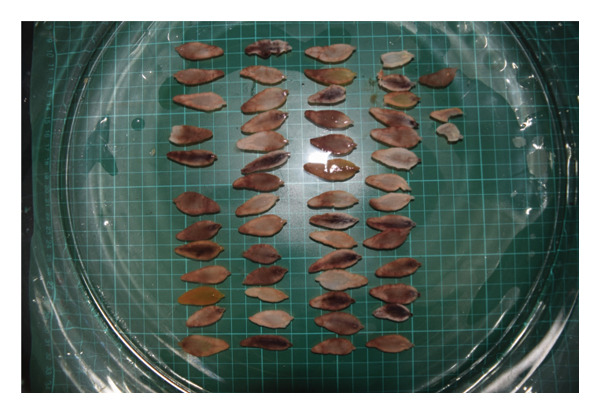
Relaxed *Fasciola* spp. recovered from ruminant host livers arranged on the stereo microscope for morphometric measurement recording.

Eight hundred and seven adult liver flukes were found in the livers of 18 goats (nine young and nine adult), 21 sheep (seven young and 18 adult), and one cattle (one young) in KRB during 2018–2020 (Figure 2). Counts and morphometric data of adult *Fasciola* spp. from each animal are shown in Table 2. The morphological patterns observed were short, broad, and curvy lancet shaped flukes that resemble previously reported *F. hepatica* found in small ruminants [20].

**TABLE 2:** Morphometric data of liver flukes from host ruminants in KRB arranged by host and age group: young (1–3 years old) and adult (4 years old and above).

No	Host	Host count	Age group	Liver fluke (*n*)	*Fasciola* spp. (мм^1^)				
					BL (min–max)	BW (min–max)	BL/BW	VS^2^‐P^3^ (min–max)	Vit^4^‐P (min–max)
1	Goat	9	Young	261 (17–46)	23.8 (12–32)	7.7 (3.8–10)	3.0	17.4 (9.8–25)	5.5 (1.8–8)
2	Goat	9	Adult	85 (2–11)	25.8 (20–32)	8.6 (6–10)	3.0	19.2 (15–25)	5.8 (5–8)
3	Sheep	7	Young	118 (6–39)	25.8 (21–31)	8.4 (7–9.3)	3.0	20.6 (14–24)	5.8 (5–7.5)
4	Sheep	14	Adult	223 (2–58)	26.0 (23–30)	8.5 (8–9.8)	3.0	21.6 (20–25)	6.3 (5–7)
5	Cattle	1	Young	120	22.4 (12–31)	7.2 (3.6–11)	3.1	21.6 (20–25)	6.3 (5–7)
Total				807	(18–32)				

^1^Millimeters.

^2^Ventral sucker.

^3^End of body.

^4^Vitelline gland.

Comparing the infection intensities, per animal, by age group, counted by liver flukes, higher intensity (*n* = 17–46 from individual) is more commonly found in young goat than other age groups and animals (Table 1). Of the *Fasciola* spp. from the cattle, 86% (103/120) were smaller (BL = 12–18 мм and BW = 3.6–5.8 мм) than liver flukes isolated from sheep and goat.

### 3.2. Coprological Analysis

**FIGURE 3:**
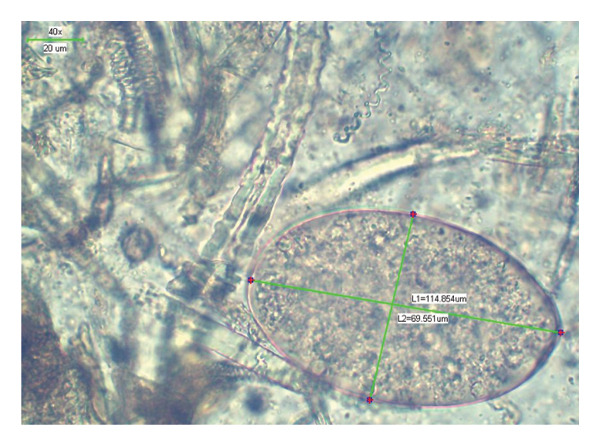
A *Fasciola* spp. egg from a liver fluke viewed with a stereo microscope with measurement bars at 40x magnification.

Liver fluke eggs, found in fecal samples, were collected from ruminants in KRB (Figure 3). A total of 100 *Fasciola* spp. eggs were measured. The trematode eggs were large, thin shelled with operculum and yellow colored, and measured 65.04–70.7 × 80.6–114.8 μm.

In Table 3, the presence of *Fasciola* spp. eggs from liver flukes of the at‐risk population was assessed using coprological analysis. *Fasciola* spp. eggs were present in 56%, 32%, and 34% of fecal samples in 2018, 2019, and 2020, respectively.

**TABLE 3:** The prevalence of *Fasciola* spp. eggs in fecal samples by host ruminants and host age from 2018 to 2020.

Host	Host age	Host count	Fascioliasis (+)	Prevalence (%)	Prevalence (%)
Goat	Young	100	38	38.0	32.8
	Adult	50	12	24.0	

Sheep	Young	120	64	53.3	48.3
	Adult	60	23	38.3	

Cattle	Young	10	8	80.0	70.0
	Adult	10	6	60.0	

### 3.3. Intermediate Host of *Fasciola* spp.

The morphometric data of the snail shells were identified as *Radix bactriana* (547/577; 94.8%) and *Lymnaea stagnalis* (30/577; 5.2%).

## 4. Discussion

This is the first report of fascioliasis in indigenous animals in Mongolia, which can pose a risk to the livestock population detrimentally impacting nomadic herders. Nomadic animal husbandry relies on livestock productivity to sustain the economic livelihoods of herders [23]. In the KRB, where a large majority of the land is used for land grazing, the detection of fascioliasis should be continually monitored [14]. This study tested for fascioliasis from October to December, potentially leading to an underestimation of the true prevalence as there is a higher risk for infection in the spring due to grazing habits of livestock and temperature [24]. Incorporating longitudinal monitoring of snails will allow for the evaluation of climatic shifts, intervention effectiveness, and habitat changes [25]. Additional studies into the epidemiology and distribution of *Fasciola* spp. throughout Mongolia would allow for public health interventions to support the livestock health of pastoralists.

The two common flukes, *F. hepatica* and *F*. *gigantica*, vary in geographic range and distribution. *F. hepatica* is typically found in temperate areas and is more widespread than *F. gigantica* which commonly inhabits tropical areas of southern and southeast Asia as well as northern Africa [26, 27]. A wide range of morphologies of *Fasciola* spp. have been reported across Japan, Taiwan, the Philippines, and Korea [28]. While it is important to differentiate between *Fasciola* spp. to determine proper epidemiological and control methods, the overlapping distribution previously reported in Asia and lack of availability of immunological tests restricted us from identifying the specific strain present in this study [15, 29–31].

Given the lack of previous data regarding *Fasciola* spp. in Mongolia, continued surveillance should be implemented in high risk areas identified through environmental analysis. With ideal environments for snail development in warmer temperatures and slow moving lakes or rivers, the detection of intermediate hosts in the KRB could be indicative of more widespread *Fasciola* spp. in this region [14]. The livestock of nomadic pastoralists which are the ruminant hosts for *Sciolala* spp. travel for pasture and water and are therefore present throughout the country. This is particularly concerning in areas where snails or ideal snail habitats are found near grazing livestock or pastures [32]. These high‐risk areas can be a risk to human health through ingestion of contaminated water or food, which can be mitigated with community education [33]. Using a One Health surveillance approach that incorporates livestock serology, snail detection, human infection, and environmental measurements is necessary to identify hotspots, provide recommendations for evidence‐based interventions, and predict shifts in *Fasciola* risk due to changing environmental conditions [34].

*Fasciola* spp. eggs were obtained from local animal fecal samples in Mongolia for the first time. The morphometric patterns of the egg, including large size, brownish yellow, with operculum and embryo cell filled internal space, oval to round shaped, indicate that it is *Fasciola* spp. Although the color, shape, shell, and internal structure of eggs are clear, previous studies have noted that due to the host species and geographical conditions, the dimensions of the eggs are not exactly uniform [20]. The size of *Fasciola* eggs found in the ruminants from the KRB was relatively small compared with measurements from Beugnet et al., 2008 [35]. Some eggs were round shaped, likely due to infection resistance of local animals and the extreme climatic conditions in the KRB. According to the sustained infection prevalence in the risk group, 32.8%–70%, between 2018 and 2020, fasciolosis may spread endemically in the targeted area of Mongolia. There are many potential confounders to these estimates including overlapping grazing areas and shared water sources. Communal grazing among pastoralist communities may lead to repeated exposure among herds sharing the same area potentially inflating prevalence or through shared water sources or other modes of transmission [36].

As the first attempt to analyze the phenotype of lymnaied snails, the intermediate host of *Fasciola* spp., in Mongolia, we identified mostly *R. bactriana* followed by *L. stagnalis*. As mentioned in the taxonomic work of the freshwater snail of Mongolia, we identified the intermediate host of fasciolosis using the dimensional data of the structure of snails [37]. The *R. bactriana* snail is a common snail in the Kharaa, Eroo, and Selenge rivers and in the depression of the Great Lakes of Mongolia [37]. Previous research shows that the *R. bactriana* snail is widely distributed in parts of Asia, Europe, and the Americas, likely explaining the high percentage of freshwater snails collected in this study [38]. Both *R. bactriana* along with other *Radix* spp. and *L. stagnalis* have been detected in Kazakhstan. Interestingly, in Kazakhstan, *L*. *stagnalis* was one of the most common snail species found in all drainage basins [39].

Given the high prevalence of *Fasicola* spp. detected in this study, especially among cattle, and the detection of intermediate hosts, there are control strategies that could be implemented Mandal, Selenge. Pastoralists could use of drugs that kill liver flukes, including triclabendazole; however, there are concerns of resistance developing [40]. Many new drugs against *Fasciola* spp. are being developed to combat this resistance [41]. Other strategies for control include grazing management such as rotating pastures, preventing feeding of wet pasture during high risk periods, and quarantine of infected animals [41]. To inform these control programs, models can be developed for the specific region to forecast disease threat [42].

This study relied on morphological differentiation of species; however, there is no measurement standardization in the literature; therefore, these results cannot be compared with external case reports [28]. Future studies need to include molecular confirmation of *Fasciola* spp. through PCR or sequencing [42]. Another limitation is the development of these flukes in two different host species, which can result in size variation in adult flukes, as growth is determined by their initial conditions [28, 43]. As a preliminary investigation into the geographic range of the snail intermediate host, our surveillance including 18 locations does not fully represent the distribution throughout Mongolia.

## 5. Conclusion

A high prevalence of fascioliasis in livestock poses a potential zoonotic risk to humans, warranting molecular and epidemiological investigations to assess public health risk. Pastoralists can adapt grazing management practices and rotate various drugs to control *Fasciola* spp. Using a One Health approach integrating livestock, human and environmental surveillance will help understand the geographic distribution across Mongolia for the evidence‐based control of fasciolosis in the region.

## Author Contributions

Conceptualization, Lkhagvatseren Sukhbaatar; methodology, Chinchuluun Boldbaatar and Michael E. von Fricken; software, Gantuya Sambuu; validation, Nora G. Cleary and Otgonpurev Sukhbaatar; investigation, Davaajargal Tserennyam, Enkhjargal Enkherdene, Gantuya Sambuu, and Munkhjargal Tserendorj; data curation, Otgonpurev Sukhbaatar; writing–original draft preparation, Lkhagvatseren Sukhbaatar and Nora G. Cleary; writing–review and editing, Nora G. Cleary and Michael E. von Fricken; visualization, Batsukh Zayat; supervision, Lkhagvatseren Sukhbaatar and Batsukh Zayat.

## Funding

The national research project is funded by the Science and Technology Foundation of Mongolia. The foundation was funded for the project titled “Surveillance for fascioliasis No SBS 2018‐14”.

## Disclosure

All authors have read and agreed to the published version of the manuscript.

## Conflicts of Interest

The authors declare no conflicts of interest.

## Data Availability

The data that support the findings of this study are available from the corresponding author upon reasonable request.
